# Mitochondrial Transplantation Therapy Ameliorates Muscular Dystrophy in *mdx* Mouse Model

**DOI:** 10.3390/biom14030316

**Published:** 2024-03-07

**Authors:** Mikhail V. Dubinin, Irina B. Mikheeva, Anastasia E. Stepanova, Anastasia D. Igoshkina, Alena A. Cherepanova, Alena A. Semenova, Vyacheslav A. Sharapov, Igor I. Kireev, Konstantin N. Belosludtsev

**Affiliations:** 1 Department of Biochemistry, Cell Biology and Microbiology, Mari State University, pl. Lenina 1, 424001 Yoshkar-Ola, Russia; lady.stepanowa2010@yandex.ru (A.E.S.); anastasi.igoshkina@yandex.ru (A.D.I.); alyona.cherepanova@bk.ru (A.A.C.); sem_al.ru@mail.ru (A.A.S.); slav.sharapov@yandex.ru (V.A.S.); bekonik@gmail.com (K.N.B.); 2 Prokhorov General Physics Institute, Russian Academy of Sciences, Vavilov St. 38, 119991 Moscow, Russia; 3 Laboratory of Mitochondrial Transport, Institute of Theoretical and Experimental Biophysics, Russian Academy of Sciences, Institutskaya 3, 142290 Pushchino, Russia; mikheirina@yandex.ru; 4 Belozersky Institute of Physico-Chemical Biology, Lomonosov Moscow State University, 119991 Moscow, Russia; iikireev@gmail.com

**Keywords:** Duchenne muscular dystrophy, skeletal muscle, mitochondrial dysfunction, mitochondrial transplantation, lipid peroxidation, muscle force

## Abstract

Duchenne muscular dystrophy is caused by loss of the dystrophin protein. This pathology is accompanied by mitochondrial dysfunction contributing to muscle fiber instability. It is known that mitochondria-targeted in vivo therapy mitigates pathology and improves the quality of life of model animals. In the present work, we applied mitochondrial transplantation therapy (MTT) to correct the pathology in dystrophin-deficient *mdx* mice. Intramuscular injections of allogeneic mitochondria obtained from healthy animals into the hind limbs of *mdx* mice alleviated skeletal muscle injury, reduced calcium deposits in muscles and serum creatine kinase levels, and improved the grip strength of the hind limbs and motor activity of recipient *mdx* mice. We noted normalization of the mitochondrial ultrastructure and sarcoplasmic reticulum/mitochondria interactions in *mdx* muscles. At the same time, we revealed a decrease in the efficiency of oxidative phosphorylation in the skeletal muscle mitochondria of recipient *mdx* mice accompanied by a reduction in lipid peroxidation products (MDA products) and reduced calcium overloading. We found no effect of MTT on the expression of mitochondrial signature genes (*Drp1*, *Mfn2*, *Ppargc1a*, *Pink1*, *Parkin*) and on the level of mtDNA. Our results show that systemic MTT mitigates the development of destructive processes in the quadriceps muscle of *mdx* mice.

## 1. Introduction

Maintaining the quality of the mitochondrial network plays an important role in the development and functioning of muscle tissue. Indeed, pharmacological modulation of mitochondrial function has been shown to be useful in the treatment of induced and genetic myopathies and significantly improves muscle state and the quality of life of experimental animals [1,2]. Along with this, one of the discussed areas of modern biomedicine is mitochondrial transplantation therapy (MTT) based on the replacement of defective mitochondria with healthy ones. In contrast to the transplantation of xeno- and allogeneic cells, the transplantation of mitochondria does not cause a noticeable immune response, and mitochondria are able to quickly penetrate into cells and maintain their functionality in muscle cells for at least 28 days [3,4]. Penetration through tissue barriers and cell membranes has been shown even with the intranasal and intravenous administration of mitochondria [5,6]. In these cases, mitochondria were also found at a distance from the injection site, indicating the spread of organelles into targeted tissues and organs [5,6]; however, the question of the depth of their circulation in the body requires more careful study. MTT has been successfully used in models of ischemia–reperfusion, Parkinson’s disease, and Alzheimer’s disease [3,4,5,7,8]. It has been shown that the injection of healthy mitochondria (of allogeneic and autogenous origin) into the heart muscle reduces the infarct zone from 30% to 6–7% [9]. It has recently been shown that the introduction of donor mitochondria into damaged skeletal muscle contributes to the normalization of neuromuscular function. In particular, using models of acute limb ischemia induced by toxic damage (by BaCl_2_) or tourniquet applications, it was found that the systemic delivery of mitochondria enhances muscle regeneration, reduces the intensity of fibrosis, and restores muscle function [6,10]. Several mechanisms underlying the therapeutic effect of MTT are discussed. It is hypothesized that additional mitochondria in regenerating muscles may increase the overall energy production required for protein assembly. In addition, MTT can enhance mitophagy (removal of defective organelles) or promote the introduction of mitochondrial DNA into the mitochondria of recipient cells [1]. It has also been shown that allogeneic mitochondria are found predominantly in the nucleus region of muscle cells, suggesting that they are optimally located for calcium buffering and prevent calcium overload of skeletal muscle fibers, as seen in cardiomyocytes [11]. Thus, the available data suggest that the administration of allogeneic mitochondria from healthy donors can be used to improve the overall health of the mitochondrial network in the cells of recipient organisms.

Previously, it was shown that mitochondria-targeted therapy can also be used in the treatment of hereditary muscular dystrophies and, in particular, Duchenne muscular dystrophy. Indeed, the pharmacological and genetic modulation of the proteins of these organelles (including those forming ion channels) [12,13,14,15,16,17] and the activation of biogenesis [18] improve mitochondrial function and this is accompanied by a decrease in the degeneration of dystrophin-deficient muscles and facilitates the development of pathology in model animals. In this work, we assessed the prospects of using MTT, which consists of the systemic injection of allogeneic mitochondria from healthy animals, to correct the muscle function of dystrophin-deficient *mdx* mice. This approach has been found to reduce muscle degeneration and improve muscle function in this model.

## 2. Materials and Methods

### 2.1. Animals

C57BL10 male mice (wild type, WT) and dystrophin-deficient male *mdx* mice (C57BL/10ScSn-mdx) were from the Animal Breeding Facility, Branch of the Shemyakin and Ovchinnikov Institute of Bioorganic Chemistry, Russian Academy of Sciences, Russia (IBCh RAS Unique Research Device “Bio-model”, Pushchino, Russia). The animals were divided into the following groups: (1) wild-type mice treated with vehicle—incubation medium (WT, *n* = 15); (2) *mdx* mice treated with vehicle—incubation medium (*mdx*, *n* = 15); (3) *mdx* mice injected with mitochondria from WT animals resuspended in the incubation medium (*mdx +* MTT, *n* = 17).

### 2.2. Isolation of Mitochondria from Skeletal Muscles of Mice and Functional Studies

The differential centrifugation method was used to isolate mitochondria from the total quadriceps of both hind limbs of mice [19]. The Bradford assay showed 25–35 mg mitochondrial protein/mL in the resulting suspension. The rate of O_2_ consumption by mitochondria was measured using Oxygraph Plus system (Hansatech Instruments, King’s Lynn, UK) and incubation buffer containing 120 mM KCl, 5 mM NaH_2_PO_4_, and 10 mM HEPES-KOH (pH 7.4) and supplemented with 2.5 mM potassium malate + 2.5 mM potassium glutamate. Amounts of 0.3 mg mitochondrial protein/mL, 200 μM ADP, and 50 μM 2,4-dinitrophenol (DNP) were used in each assay. The rates of substrate oxidation were expressed as nmol O_2_/min per 1 mg of protein and the respiratory control ratio (RCR = state 3/state 4) was determined according to [20].

Mitochondrial Ca^2+^ transport was estimated by arsenazo III absorbance (675–685 nm) using Varioskan LUX spectrofluorimeter (Thermo Fisher Scientific, Waltham, MA, USA) [21]. Mitochondria were resuspended in 210 mM mannitol, 70 mM sucrose, 1 mM KH_2_PO_4_, 10 μM EGTA buffer at a final concentration of 0.3 mg/mL and supplemented with 50 μM arsenazo III and 10 mM HEPES-KOH buffer (pH 7.4). The total number of added Ca^2+^ pulses that caused the spontaneous release of an ion from the mitochondrial matrix due to the induction of mitochondrial permeability transition pore (MPT-pore) opening corresponded to the organelle’s calcium retention capacity (CRC).

Ca^2+^ overload was measured using the above system and incubation medium. To assess free matrix Ca^2+^, 3 mg/mL of isolated mitochondria was treated with a membrane-permeabilizing agent, 0.1 mg/mL alamethicin (ALM). The relative amount of matrix Ca^2+^ was assessed by the increase in absorbance following permeabilization.

Quantitative determination of thiobarbituric acid-reactive substances (mainly malondialdehyde (MDA)) was used to assess the intensity of lipid peroxidation in mitochondria [22].

### 2.3. Administration of WT Mouse Skeletal Muscle Mitochondria to mdx Animals and Labeling Them with MitoTracker Red Chloromethyl-X-Rosamine (CMXRos)

**Figure 1:**
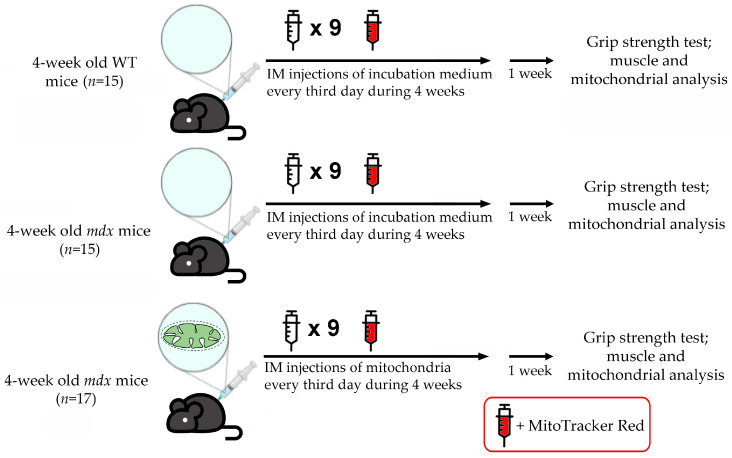
The experimental design of the study; IM: intramuscular.

Skeletal muscle mitochondria from male C57BL/10 mice isolated by differential centrifugation (Section 2.2) were administered to *mdx* animals using a tuberculin syringe with a 30-gauge needle. We used mitochondria whose respiratory control ratio was close to 4 (substrates—2.5 mM glutamate and 2.5 mM malate) (Table S1). Pre-incubation of organelles satisfying this condition with 10 μM cytochrome *c* increased the rate of respiration by no more than 15% indicating sufficient integrity of the membranes of isolated organelles [6]. Typical respiration curves of the used mitochondria and average parameters of their respiration are given in the supplementary material (Figure S1 and Table S1). Mitochondria meeting the conditions were resuspended in an incubation buffer containing 120 mM KCl, 5 mM NaH_2_PO_4_, and 10 mM Hepes-KOH (pH 7.4) and supplemented with respiratory substrates (2.5 mM glutamate, 2.5 mM malate, and 5 mM succinate) and used for further injections into *mdx* mice. In this case, each animal (*n* = 17) received two injections of the organelle suspension (per ~50 μL), one each into the muscles of the quadriceps and gastrocnemius group of both hind limbs (1 µg mitochondrial protein/g body weight in total, protein determined by Bradford assay). Control dystrophin-deficient (*n* = 15) and WT animals (*n* = 15) received injections of the specified incubation medium that did not contain mitochondria (vehicle). Injections were made every third day and each animal was subjected to 10 procedures. A brief research protocol is shown in Figure 1.

Mitochondria obtained for the last series of injections were stained using the mitochondrial potential-dependent probe MitoTracker Red CMXRos (Invitrogen, Carlsbad, CA, USA) for subsequent visualization. Mitochondria meeting the above conditions were resuspended in 120 mM KCl, 5 mM NaH_2_PO_4_, and 10 mM Hepes-KOH (pH 7.4) buffer supplemented with 1 μM MitoTracker Red CMXRos and respiratory substrates (5 mM glutamate, 5 mM malate, and 10 mM succinate) for 10 min at 4 °C in the dark. The suspension was centrifuged at 8000× *g* 10 min at 4 °C. The mitochondrial pellet was resuspended in the incubation medium and washed three times by centrifugation at 8000× *g* for 10 min to remove the unbound fluorescent dye. The supernatant after the third wash was checked for the presence of the dye signal (excitation 579 nm and emission 599 nm) using a Varioskan LUX plate reader (Thermo Fisher Scientific, Waltham, MA, USA). In the absence of a signal, the resulting stained mitochondria were resuspended in incubation buffer supplemented with 2.5 mM glutamate, 2.5 mM malate, and 5 mM succinate and used for further injections into *mdx* mice. Control *mdx* mice received an injection of incubation medium supplemented with substrates and 1 μM MitoTracker Red CMXRos. A single injection of stained mitochondria was used to minimize the possible effects of MitoTracker Red on the parameters we assessed.

Upon completion of the series of injections, the animals were housed for a week in a vivarium.

### 2.4. Imaging of Mitochondrial Transplantation

The success of mitochondrial transplantation was assessed on freshly prepared transverse cryosections of *mdx* mice skeletal muscles (*vastus lateralis* of both hind limbs). Quadriceps of *mdx* mice treated with vehicle were used as controls. The muscles were frozen in isopentane cooled with liquid nitrogen [23] and then cut transversally into cryosections (7 µm thick) using a Minux FS800A automated cryostat (RWD, Shenzhen, China). Cell nuclei were labeled with Hoechst 33342 fluorescent dye (Thermo Fisher Scientific, excitation 352 nm; emission 454 nm), muscle fiber membranes were labeled with biotinylated lectins and streptavidin-AlexaFluor488 fluorescent reporter. Stained preparations were studied using the EVOS M5000 imaging system and DAPI, Texas Red, and GFP fluorescent cubes.

### 2.5. Grip Strength Test and Wheel-Running Activity

The grip strength test (IITC Life Science, Woodland Hills, CA, USA) was used to assess the muscle strength of the animals. During the experiment, the animal pulls a special handle (T-bar for the front or hind paws and the mesh for the four paws of the animal), which is attached to a digital force transducer. The results are presented in grams per animal body weight. Each mouse had three trials, and the average was used for the final score.

The running-wheel activity test was used to evaluate the endurance and motor activity of mice. All animals were previously (one week before) familiarized with the test environment before testing began. During the test, mice (*n* = 5 per group) were housed in a cage equipped with a running wheel (Ugo Basile, Varese, Italy) for 96 h (before withdrawal from the experiment). The average running distance over 24 h was analyzed.

### 2.6. Creatine Kinase Analysis

Mouse blood serum was spectrophotometrically analyzed for creatine kinase activity (Vector-Best, Novosibirsk, Russia).

### 2.7. Histological Studies

The quadriceps muscle (*vastus lateralis,* eight samples per group from different animals) and the gastrocnemius muscle (four samples per group from different animals) were embedded in paraffin wax after preliminary fixation in neutral buffered 10% formalin. The resulting paraffin blocks were cut using Minux S710 rotary microtome (RWD, Shenzhen, China) into 5 μm serial sections. Hematoxylin and eosin (H&E) staining protocol was used to assess the severity of histological changes in the samples. Alizarin red S staining was performed to detect calcium deposits in the quadriceps samples. All samples were examined using an EVOS M5000 imaging system (Thermo Fisher Scientific) as shown earlier [16]. All histological images (the mid-belly region of the muscles, defined according to [23]) were analyzed using ImageJ software version 1.53 (National Institutes of Health, Bethesda, MD, USA). The percentage of centrally nucleated fibers (CNF) was calculated by counting all the fibers in the H&E stained muscle cross-sections. In this case, the minimal Feret’s diameter (the minimum distance of parallel tangents at opposing borders of the muscle fiber) of all fibers was also measured. The level of tissue calcification was expressed as the % ratio of Alizarin red S staining areas to the total area of the cross-section. The average values obtained from each animal were used for statistical analysis (Section 2.11).

### 2.8. Transmission Electron Microscopy

The quadriceps muscles (*vastus lateralis,* three samples per group from different animals) were fixed, dehydrated, encapsulated in epon resin and cut into ultrathin sections (60–70 nm) as shown previously [12,16]. To control the transverse orientation of the muscle fibers and determine the mid-belly region of the muscle (according to Section 2.7), semi-thin sections were first obtained and analyzed using the EVOS M5000 imaging system (Thermo Fisher Scientific). Ultrathin sections (60–70 nm) were obtained from the epone blocks using a Leica EM UC6 ultramicrotome (Leica, Wetzlar, Germany). Visualization of samples counterstained with uranyl acetate and lead citrate was carried out using a JEM-1400 electron microscope (JEOL, Japan) based on the "Superresolution microscopy and spectroscopy" core facility at the Belozersky Institute of Physicochemical Biology. Image Tool 3.0 software was used to analyze the electron microscopy images. For morphometric analysis, a routine measurement method was used, requiring manual contouring of the cross-sections of mitochondria along their outer membrane and membranes of the sarcoplasmic reticulum (SR) associated with interfibrillar mitochondria (mitochondria-associated membranes—MAM-contacts) within 30 nm [24]. The results of morphometric analysis were presented as the ratio of the total length of the MAM-contact to the mitochondrial perimeter, expressed as a percentage. A total of 150 cross-sectional profiles (50 profiles per sample) of mitochondria were collected from each group of mice. The average values obtained from each animal were used for statistical analysis (Section 2.11).

### 2.9. RNA Extraction, Reverse Transcription, and Quantitative Real-Time PCR

Using the ExtractRNA kit (#BC032, Eurogen, Moscow, Russia), the total RNA was prepared from 100 mg of deep-frozen quadriceps samples (ten samples per group from different animals). The real-time PCR was performed using the qPCRmix-HS SYBR reaction mixture (Eurogen, Moscow, Russia) and DTLite5 amplifier (DNA-Technology LLC, Moscow, Russia). Primer-BLAST was used to select and analyze gene-specific primers [25] (Table 1). A comparative C_T_ method was used to assess the relative expression level of each gene [26].

### 2.10. Mitochondrial DNA Quantification

An amount of 1 ng of the total DNA (nuclear and mtDNA) isolated form 10 mg of quadriceps muscle (ten samples per group from different animals) using the DNA-Extran 2 kit (Sintol, Moscow, Russia) was taken for the reaction. The mtDNA content in the samples was assessed by PCR [27]. The results were expressed as the mtDNA/nuclear DNA ratio by measuring the expression of the *ND4* gene of the mitochondrial genome and the nuclear-encoded GAPDH gene. The primers used are shown in Table 1.

### 2.11. Statistical Analysis

GraphPad Prism 8.0.1 software was used for data analysis. Results are expressed as mean ± SEM. The statistical significance of the differences between the groups was evaluated using one-way analysis of variance (ANOVA) followed by the Tukey multiple comparison post hoc test. A value of *p* < 0.05 was chosen for statistical significance.

## 3. Results

### 3.1. Visualization of MTT and Its Effect on the Skeletal Muscle Ultrastructure of mdx Mice

**Figure 2:**
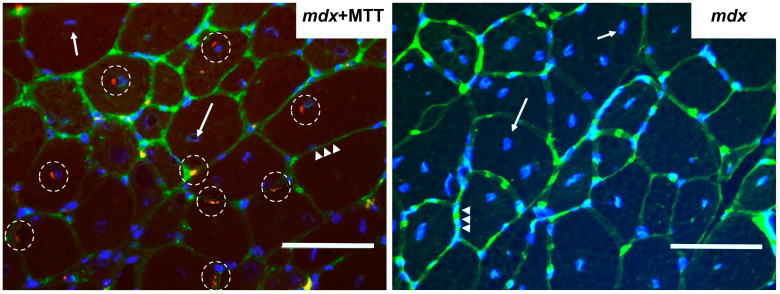
Representative micrographs of quadriceps tissue from *mdx* mice treated with allogeneic mitochondria labeled with MitoTracker Red CMXRos and *mdx* mice injected with vehicle (negative control). The blue color corresponds to cell nuclei stained with Hoechst 33342 fluorescent dye (indicated by arrows). Muscle fiber membranes were stained with biotinylated lectins and streptavidin-AlexaFluor488 fluorescent reporter (indicated by triangles). Red clusters (left image, indicated by dotted circles) correspond to mitochondria stained with MitoTracker Red CMXRos. Scale bar is 75 μm.

It has been previously demonstrated that mitochondria injected into skeletal muscles successfully penetrate into the muscle fibers of the recipient [10]. In this work, we also assessed the success of MTT after 10 injections of allogeneic mitochondria into the skeletal muscles of dystrophin-deficient mice. For this, serial transverse cryosections were prepared from the freshly isolated muscles (quadriceps of both hind limbs) of experimental groups of mice. MitoTracker Red CMXRos fluorescence signals corresponding to pre-stained allogeneic mitochondria were assessed using fluorescence microscopy. One can see that the muscle fibers of *mdx* mice injected with labeled mitochondria show focal probe fluorescence (Figure 2), while no intense fluorescent signal was detected in the muscles of control *mdx* animals that received the vehicle (incubation medium, including the last injection containing the dye). We noted the localization of labeled mitochondria both in the endomysium and in various parts of muscle fibers (for example, in the subsarcolemmal and perinuclear spaces). At the same time, we also do not exclude the distribution of part of the probe into the mitochondrial apparatus of recipient animals. In the case of control *mdx* mouse muscles, MitoTracker Red CMXRos appears to be evenly distributed throughout the tissues.

**Figure 3:**
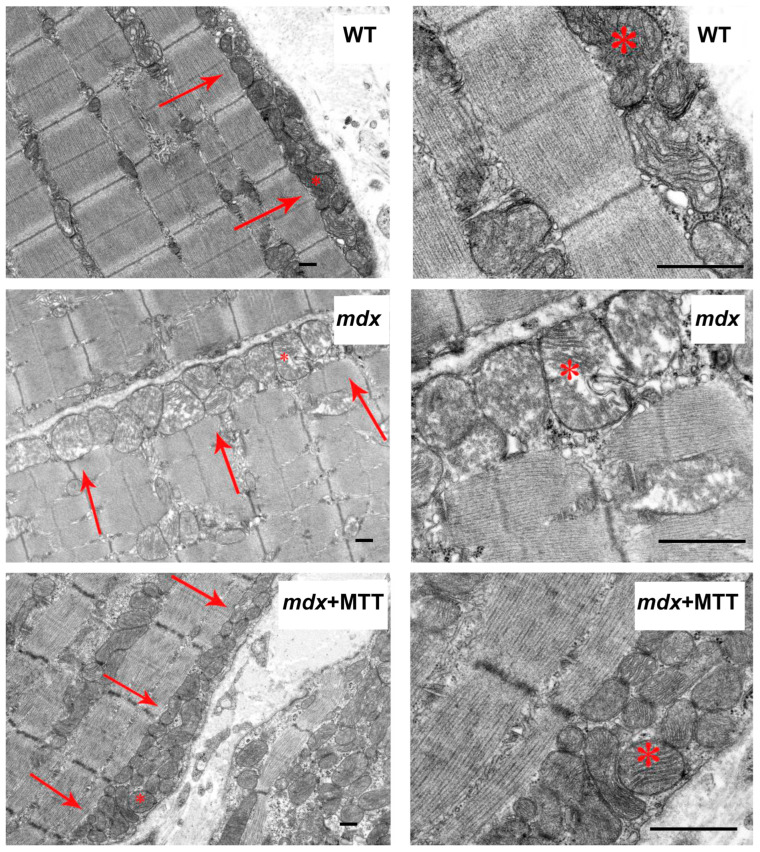
Representative transmission electron micrographs of mouse quadriceps sections. Mitochondria of the subsarcolemmal population are highlighted with red arrows. The asterisk marks the same mitochondria at low (**left column**) and high (**right column**) magnification. Scale bar is 1 μm.

**Figure 4:**
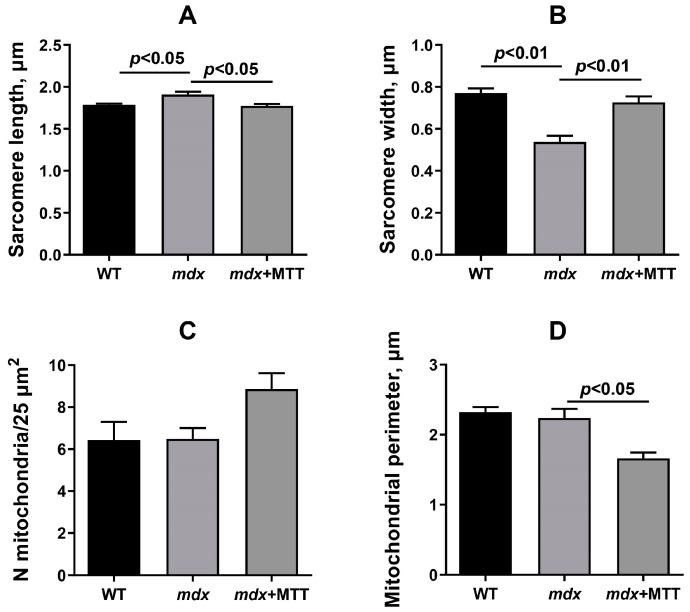
Electron micrograph (Figure 3) profiles: sarcomere length (**A**); sarcomere width (**B**); number of mitochondria per plate (**C**); and mitochondrial perimeter (**D**). The number of examined fields of view in the groups varies from 40 to 50. The data are presented as means ± SEM (*n* = 3).

We have evaluated the skeletal muscle ultrastructure of the experimental groups of mice using electron microscopy. Figure 3 shows that skeletal muscle myofibrils of mice in the WT group are characterized by classical architecture. The subsarcolemmal region, located on the periphery of the muscle fiber, contained small accumulations of mitochondria. The organelles were spherical or elongated. There were no disturbances in the location of the cristae or damage to the outer or inner membranes. Mitochondria with a dark matrix and densely packed cristae predominated. The sarcoplasmic reticulum was mainly represented by flattened cisterns. The muscle fibers of the skeletal muscles of the *mdx* demonstrated mosaically arranged destructive changes in myofibrils, sarcoplasm, sarcoplasmic reticulum, and mitochondria. In a number of cases, we observed a thinning of the diameter of myofibrils without disturbing the organization of sarcomeres, a curvature of the Z-line, and an expansion of the sarcoplasmic spaces between myofibrils. The sarcomeres of *mdx* mice showed a significant decrease in width compared to WT mice and an increase in their length (Figure 4B), which corresponds to the known data [28]. The sarcoplasm showed an increase in the content of glycogen, secondary lysosomes, and the expansion and fragmentation of the sarcoplasmic reticulum to bubbles of different diameters. In the subsarcolemmal region, mitochondria were located in small clusters, as well as in the WT group. However, there were fundamental differences in the ultrastructure of the mitochondria. Significantly swollen spherical mitochondria predominated, containing destroyed cristae and zones of sharp matrix clearing; cracks and ruptures were observed in the outer membrane of some organelles.

The *mdx* group of animals treated with mitochondrial injections (*mdx* + MTT group) retained sarcoplasmic extensions between myofibrils with increased glycogen content and fragmented sarcoplasmic reticulum (Figure 3). We observed the recovery of sarcomere size to the level of WT animals (Figure 4A,B). This group, in contrast to the *mdx* and WT groups, was characterized by large accumulations of mitochondria in the subsarcolemmal region. The *mdx* + MTT group showed a mitochondrial structure similar to the WT group. Mitochondria were spherical or elongated with a dark matrix and densely packed cristae. A distinctive feature of the *mdx* + MTT group was a tendency to increase the number of small mitochondria with a small number of cristae in the subsarcolemmal region (Figure 4C,D).

**Figure 5:**
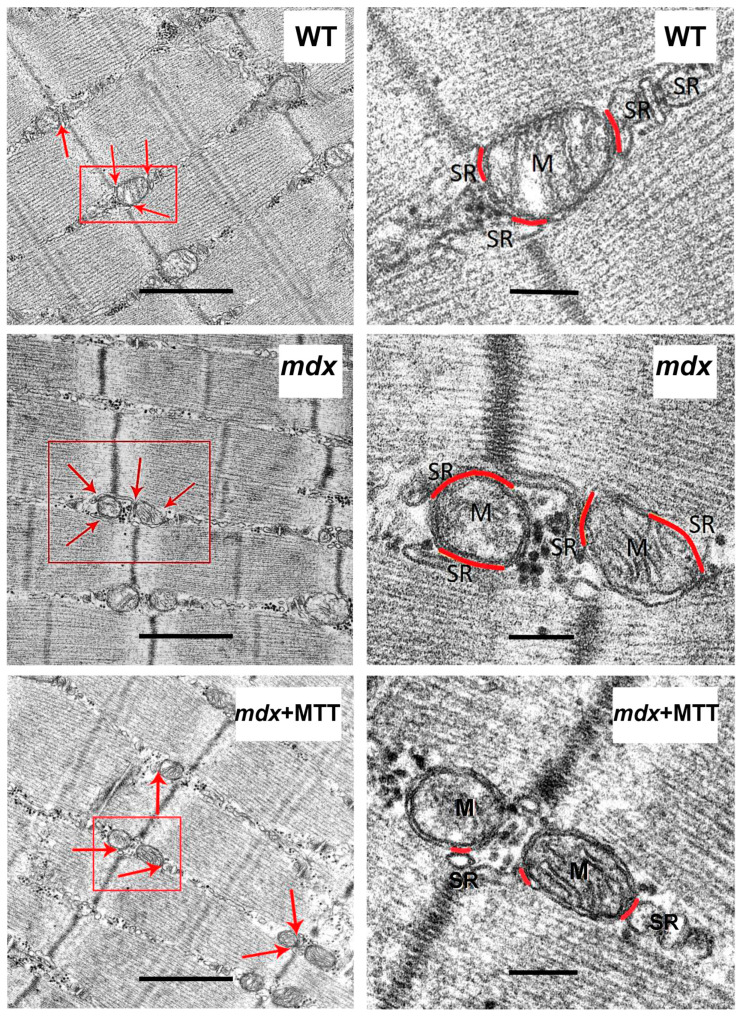
Representative transmission electron micrographs of mouse quadriceps sections. (**Left**) panels show images at low magnification, and (**right**) panels at high magnification. Red arrows indicate the SR/mitochondria (M) contact sites. The red frame highlights the area shown at the corresponding high magnification. The red line on the right panels marks the MAM-contact. Scale bar is 1 μm (**left column**) or 200 nm (**right column**).

**Figure 6:**
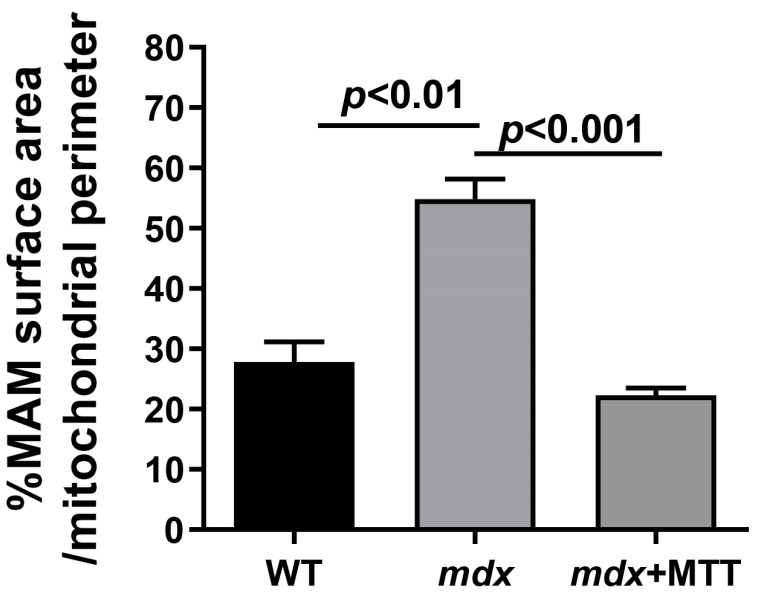
Percentage of MAM surface area per mitochondrion perimeter in each microscopic field. A total of 150 cross-sectional profiles of mitochondria (50 profiles per sample) were selected for analysis in each group of animals. The data are presented as means ± SEM (*n* = 3).

We also analyzed the state of mitochondria-associated membranes (MAM-contacts) in experimental samples, which are formed between the SR and mitochondria (mainly interfibrillar). These structures play an important role in the dynamic regulation of Ca^2+^ homeostasis and the functional activity of mitochondria [24,29]. Figure 5 shows that the ultrastructure of interfibrillar mitochondria does not differ between experimental groups of animals. On the other hand, we noted changes in SR/mitochondria contact interactions. Indeed, the quantitative data summarized in Figure 6 show that the % of MAM surface area per mitochondrion perimeter in *mdx* mice increases by two times compared with the WT group, indicating an increase in the formation of MAM-contacts in dystrophin-deficient muscles. At the same time, *mdx* + MTT mice demonstrate a significant decrease in the percentage of MAM surface area per mitochondrion perimeter compared to *mdx* animals.

### 3.2. Effect of MTT on Skeletal Muscle Degeneration and Muscle Strength in mdx Mice

Based on the results obtained, one could assume that MTT improves mitochondrial ultrastructure, SR/mitochondria interactions, and sarcomere size in *mdx* muscles. We assessed the effect of MTT on disease progression in *mdx* mice and on the intensity of degeneration/regeneration cycles, tissue calcification, creatine kinase leakage, and, finally, on the muscle function of mice.

**Figure 7:**
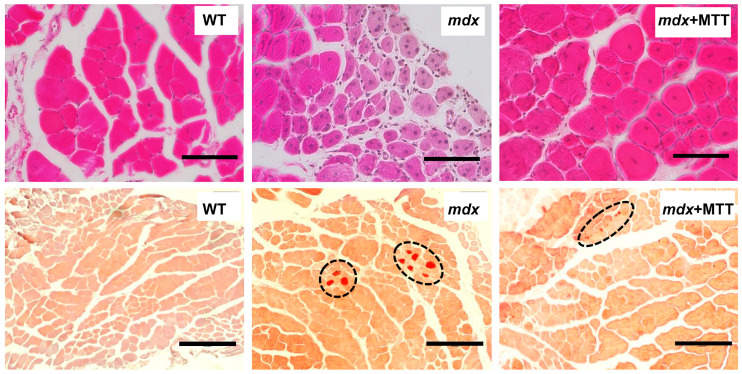
Representative histology images of quadriceps muscles showing CNF (top line, H&E staining) and calcified area (bottom line, indicated by dotted circles, Alizarin red staining). Scale bar is 75 μm (**top line**) and 300 μm (**bottom line**).

**Figure 8:**
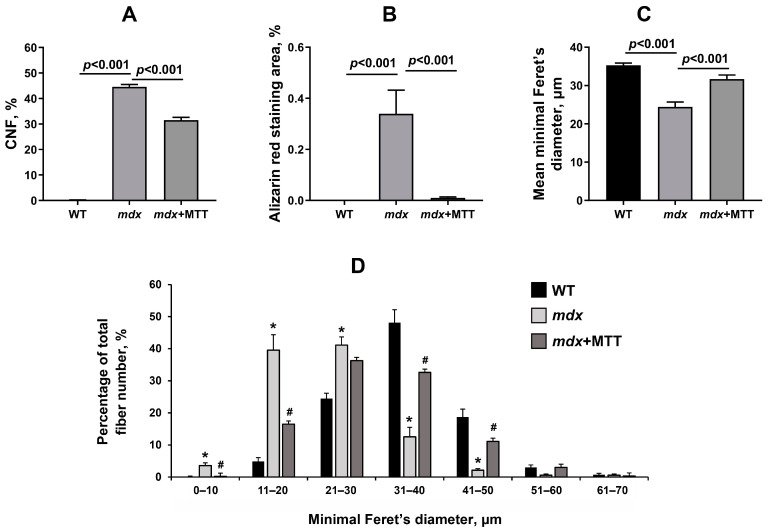
The percentage of CNF (**A**), Alizarin red staining area (**B**), mean minimal Feret’s diameter (**C**), fiber size distribution (**D**), % of the total fiber number) in the quadriceps of experimental animals. The data are presented as means ± SEM (*n* = 8). * *p* < 0.001 (versus WT group), # *p* < 0.001 (versus *mdx* group).

We scored CNF in the H&E stained samples in all groups to examine the intensity of degeneration/regeneration cycles. The quadriceps of *mdx* mice show extensive CNF reflecting ongoing muscle degeneration and regeneration (Figure 7 and Figure 8A). A similar picture is observed in the case of the gastrocnemius muscle (Figure S2). Using Alizarin red staining, we also confirmed an increase in the level of tissue calcification in *mdx* mice (Figure 7 and Figure 8B). In addition, we noted a decrease in the mean minimal Feret’s diameter of muscle fibers in *mdx* mice (Figure 8C). In *mdx* mice, smaller fibers predominated (Figure 8D), which is characteristic for newly formed muscle fibers during repeated cycles of regeneration and degeneration [30]. MTT resulted in a significant decrease in the first two parameters, as well as increase in the average diameter of muscle fibers. The latter is due to a decrease in the number of small fibers (11–20 µm) and an increase in the number of large fibers (mainly 31–50 µm). However, it should be noted that muscle fiber distribution in the *mdx* + MTT group is not normalized to WT sizes (there is a shift towards smaller fibers compared to WT mice). MTT was also accompanied by a decrease in central nucleation in the gastrocnemius of *mdx* mice (Figure S2); however, the effect was less pronounced compared to that of the quadriceps (1.26-fold vs. 1.41-fold) and in general, the gastrocnemius muscles of this group show a dystrophic phenotype, which in this case indicates a weak effect of MTT.

**Figure 9:**
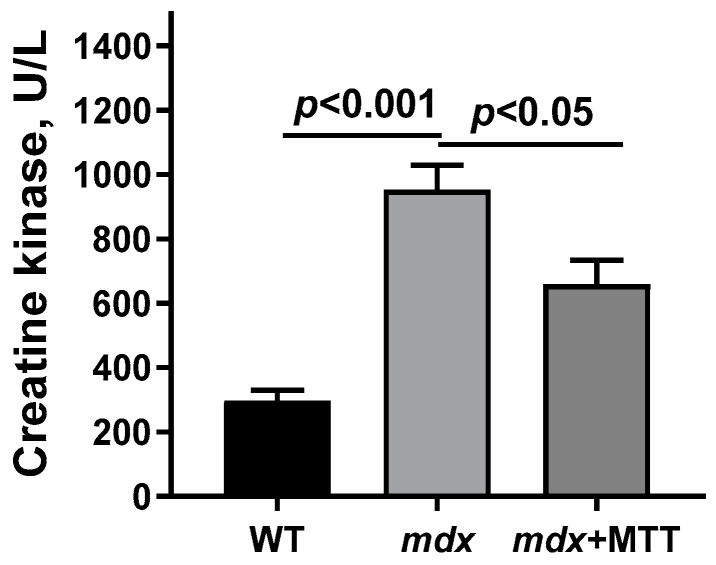
Creatine kinase activity in the blood serum of experimental animals. The data are presented as means ± SEM (*n* = 8–12).

One of the main diagnostic criteria reflecting the development of DMD is a high level of creatine kinase in the blood serum. As previously shown in [12] and confirmed here, *mdx* mice have increased levels of serum creatine kinase activity compared to WT mice (Figure 9), indicating enzyme leakage and muscle fiber membrane rupture. In this case, *mdx* mice injected with allogeneic mitochondria show a significant reduction in serum enzyme levels compared to control *mdx* animals.

**Figure 10:**
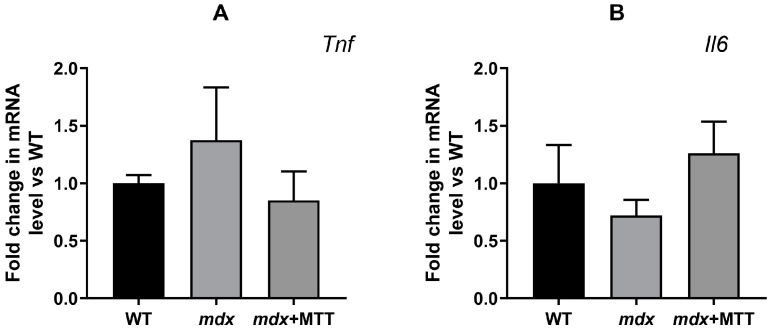
Effect of MTT on the relative mRNA levels of *Tnf* (**A**) and *Il6* (**B**) in the quadriceps of experimental animals. The data are presented as means ± SEM (*n* = 5).

It is important to note that MTT had no significant effect on the expression of the *Tnf* and *Il6* genes, encoding the proinflammatory cytokines tumor necrosis factor-α and interleukin-6, respectively (Figure 10). This may indicate the absence of a significant immune and inflammatory response to injections of mitochondria into muscle tissue, which is consistent with data from other groups [4,6]. It should be noted that we did not detect changes in the mRNA levels of these cytokines in the quadriceps of *mdx* mice, which contradicts the data obtained on older *mdx* mice [31,32], but corresponds to the parameters of young *mdx* mice [33]. It should also be noted here that this may be due to the effect of components of the incubation medium administered to the animals, and in particular to energy substrates (glutamate, malate, succinate), which are known to modulate the inflammatory response [34,35].

**Figure 11:**
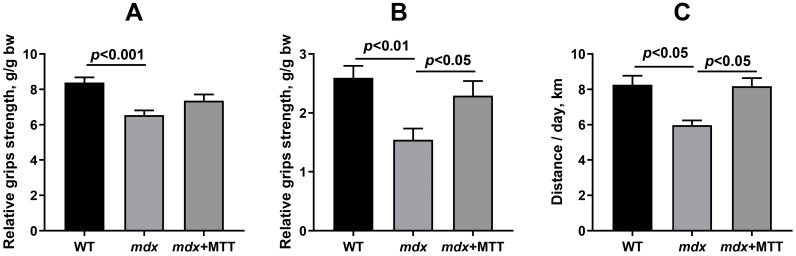
Effect of MTT on muscle strength and motor activity in mice. (**A**) Grip strength test results obtained with a mesh (the animal uses four paws). (**B**) Grip strength test results obtained using a T-bar (the animal uses only the hind limbs). The data are presented as means ± SEM (*n* = 15–17). (**C**) The average distance (km) traveled per 24 h on all 96 h of running wheel free access. The data are presented as means ± SEM (*n* = 4).

In addition, we assessed the muscle strength of the mice using the grip strength test, as well as the motor activity of the animals using activity wheels. In the present experiment, we used a mesh to assess the function of all paws of the animal, as well as a T-bar to assess the function of the hind limbs that received mitochondrial injections. One can see that dystrophin-deficient mice show low levels of muscle strength compared to WT animals, which is consistent with previous data [12,16]. This applies both to the test involving the use of all the paws of the animal, and to that involving only the hind limbs (Figure 11A,B). In this case, *mdx* mice treated with mitochondrial injections show a trend towards increased muscle strength when using four paws (Figure 11A). At the same time, when assessing the muscle strength of the hind limbs of animals subjected to the injection of allogeneic mitochondria, the differences were significant and indicated the normalization of this parameter (Figure 11B). It can also be seen that MTT leads to an increase in motor activity in *mdx* animals, as evidenced by data obtained using activity wheels (Figure 11C).

### 3.3. Effect of MTT on the Functioning of Skeletal Muscle Mitochondria in mdx Mice

We also evaluated the parameters of mitochondrial function in *mdx* mice injected with allogeneic organelles. To do this, we isolated mitochondria from the skeletal muscles (quadriceps muscles of both hind limbs) of mice and evaluated their functional parameters: efficiency of respiration and oxidative phosphorylation; calcium loading and retention capacity, reflecting resistance to MPT-pore opening; and lipid peroxidation intensity in organelles. Table 2 shows that the quadriceps muscle mitochondria of *mdx* mice demonstrate a reduction in the rate of ADP-stimulated respiration (state 3) and the RCR parameter compared to the mitochondria of WT animals. These results are consistent with previously obtained data [12,16]. The mitochondria of animals of the *mdx* + MTT group show a further decrease in the rate of respiration in state 3 and the RCR. Along with this, we also noted a decrease in the rate of uncoupled respiration in the 3U_DNP_ state (in the presence of the protonophore uncoupler 2,4-dinitrophenol (DNP) causing maximal stimulation of respiration).

**Figure 12:**
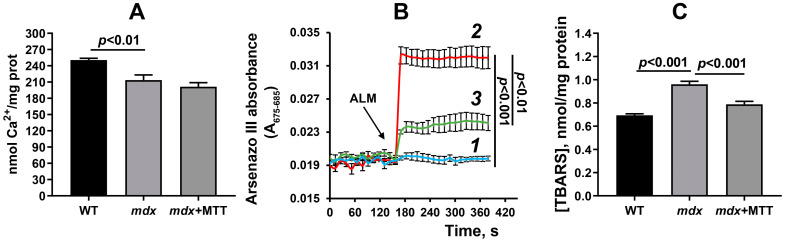
Functional parameters of skeletal muscle mitochondria. (**A**) Calcium retention capacity of isolated quadricep mitochondria. The results are presented as means ± SEM (*n* = 8). (**B**) Mitochondrial Ca^2+^ load assay from quadricep of the WT (curve *1*), *mdx* (curve *2*), and *mdx* + MTT (curve *3*) mice. An amount of 0.1 mg/mL alamethicin (ALM) was used. The results are presented as means ± SEM (*n* = 5). (**C**) The level of MDA products in the quadricep mitochondria. The results are presented as means ± SEM (*n* = 8).

DMD is accompanied by increased sensitivity of skeletal muscle mitochondria to calcium-dependent MPT-pore opening [12,14,16,22]. This is manifested in a decrease in the calcium retention capacity of organelles in dystrophin-deficient mice compared to WT animals (Figure 12A). Mitochondria from *mdx* + MTT mice are not different from control *mdx* animals with dystrophin deficiency. At the same time, we also observed that mitochondria from the quadriceps of *mdx* mice released more Ca^2+^ than WT mitochondria in response to the addition of the permeabilizing agent alamethicin, which suggests that isolated dystrophic mitochondria are in a state of Ca^2+^ overload, and MTT reduces this overload (Figure 12B).

In addition, mitochondrial dysfunction in DMD is known to be accompanied by oxidative stress and, in particular, increased levels of MDA (the end product of lipid peroxidation) in skeletal muscle mitochondria [12,16]. We have obtained similar results in the present work (Figure 12C). In this case, the mitochondria of mice of the *mdx* + MTT group show a reduction in the level of MDA products compared to control *mdx* mice, which may indicate a decrease in the intensity of lipid peroxidation and oxidative stress in the skeletal muscles of this group of animals.

### 3.4. Effect of MTT on Expression of Mitochondrial Signature Genes

**Figure 13:**
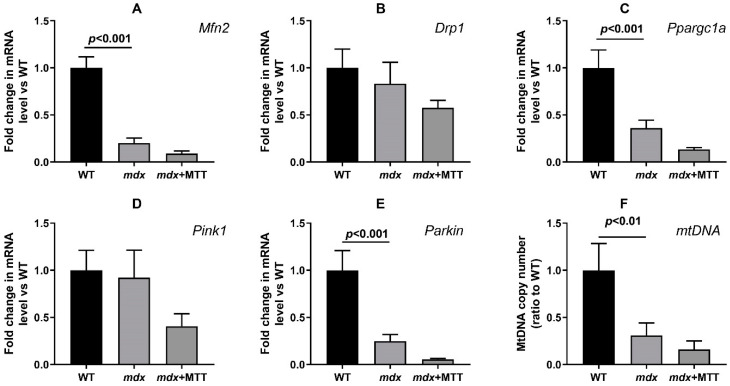
Effect of MTT on the relative mRNA levels of *Mfn2* (**A**), *Drp1* (**B**), *Ppargc1a* (**C**), *Pink1* (**D**), *Parkin* (**E**), and mtDNA (**F**) in the quadriceps of experimental animals. The data are presented as means ± SEM (*n* = 10).

Dystrophin-deficient muscles show profound changes in the systems responsible for mitochondrial fusion/fission, mitochondrial biogenesis, and mitophagy [12,18,36]. Skeletal muscles of *mdx* mice show reduced expression of the *Mfn2*, *Ppargc1a*, *Pink1*, and *Parkin* genes (Figure 13). This may indirectly indicate a decrease in the intensity of the processes concerned with mitochondrial fission and organelle biogenesis, as well as mitophagy controlled by proteins that are encoded by the corresponding genes. The expression of the *Drp1* gene responsible for mitochondrial fission does not change in dystrophic muscle. All this is also accompanied by a significant decrease in the level of mtDNA. One can see that MTT has no effect on the level of these genes and the mtDNA in the skeletal muscles of *mdx* mice.

## 4. Discussion

The development of DMD is known to be accompanied by a dramatic change in the mitochondrial ultrastructure, a reduction in the efficiency of oxidative phosphorylation, and the development of oxidative stress contributing to inflammation, intense muscle tissue degeneration, and tissue calcification. All these phenomena are also observed in model animals and, in particular, in *mdx* mice and make a significant contribution to the progression of the DMD pathology, leading to a decrease in muscle strength and endurance in animals [12]. However, it is important to note that *mdx* mice have a relatively mild phenotype compared to wild-type animals and that they do not recapitulate the fibro-fatty progression observed in patients.

Mitochondrial dysfunction in DMD can be mitigated or eliminated by the use of specific pharmacological agents capable of modulating the activity of mitochondrial targets. These targets include the proteins of mitochondrial membranes involved in the regulation of Ca^2+^ and K^+^ homeostasis, as well as the production of ROS in organelles. In particular, an increase in the Ca^2+^ buffering capacity of mitochondria by MPT-pore blockers cyclosporin A and its analog alisporivir (or Debio025) or isoxazoles (like TR001) leads to the normalization of mitochondrial function and alleviates the pathology of skeletal and cardiac muscles [12,13,14,15,28]. Promising is the activation of potassium fluxes in mitochondria contributing to the reduction in oxidative stress and the development of destructive processes in muscle tissues [16]. Recently, a positive effect of the inhibition of VDAC channels in the outer membrane of mitochondria was revealed, based on a decrease in the generation of ROS by mitochondria [17]. Another approach is associated with the knockout of genes encoding specific mitochondrial proteins. In particular, the knockout of cyclophilin D (MPT-pore regulatory protein) is known to improve mitochondrial function and slow the rate of muscle fiber degeneration in *mdx* mice [13]. Recently, using a model of δ-sarcoglycan-deficient muscular dystrophy, it has been shown that a therapeutic effect can be achieved by knocking out the adenylate translocator (ANT) (a possible channel component of the MPT-pore) [37], whose level is also elevated in the skeletal muscles of *mdx* mice [22].

In this work, we applied a fundamentally different approach associated with MTT. Intramuscular administration of allogeneic mitochondria from healthy animals to dystrophin-deficient mice has been found to alleviate skeletal muscle pathology. This is manifested in a decrease in the intensity of destructive processes in muscle fibers, as evidenced by the normalization of the size of fibers and sarcomeres, CNF reduction, as well as the level of calcium deposits in the skeletal muscles of *mdx* + MTT animals (Figure 7 and Figure 8). Importantly, MTT had no effect on the expression of *Tnf* and *Il6* genes in dystrophin-deficient quadriceps, reflecting the inflammatory status of the muscle tissue (Figure 10), which may indicate a lack of immune response. This is generally consistent with data from other groups [4,6]. However, it should be noted that there is evidence of an anti-inflammatory effect of MTT in a number of other pathological models [38,39].

All these effects are also accompanied by a decrease in the level of creatine kinase in the blood serum of *mdx* mice (Figure 9), which may indirectly reflect the preservation of the integrity of muscle fibers. However, further studies are needed to evaluate the effect of MTT on membrane stability and the level of cell surface molecules, reflecting the integrity of the myofibers of the recipient muscles (in this case, the quadriceps and gastrocnemius). Indeed, it is known that the endoplasmic reticulum stress response increases the trafficking of cell adhesion complexes to the sarcolemma in *mdx* and δ-sarcoglycan-deficient mice without the delivery of genetic vectors for these specific complexes [40]. This cannot be excluded in the case of MTT, which affects SR/mitochondria contact interactions.

In this work, we also noted an improvement in the muscle strength of animals in the grip strength test (Figure 11). However, in this case, it can be assumed that the effect is local, since we noted a significant improvement in muscle function only in the hind limbs of *mdx* mice treated with mitochondria. However, we cannot exclude that this effect may be due to the administered dose of mitochondria and the method of their administration (intramuscular). On the other hand, it can be seen that MTT leads to a significant increase in the motor activity of *mdx* animals (Figure 11C), which indicates an improvement in the quality of life of experimental animals. However, it should be noted that wheel activity test is voluntary and it is not equally reliable as ‘forced’ tests.

One could assume that the effect of MTT is based on the improvement of the mitochondrial population in the skeletal muscles of *mdx* mice. The injected mitochondria were visualized on cryosections of the skeletal muscles of recipient *mdx* mice a week after the completion of the series of mitochondrial injections (Figure 2). It should be noted that there are currently many speculations about the mechanisms of mitochondrial transfer and they need to be further investigated [41,42]. Recent evidence shows that injured muscle fibers in vivo recruit donor mitochondria better than non-injured ones [6]. Therefore, the presence of microtears, characteristic of the sarcolemma of dystrophin-deficient muscles [43], may be a factor contributing to the successful delivery of donor mitochondria to muscle fibers. 

One can see that MTT led to an improvement in the ultrastructure of the subsarcolemmal population of skeletal muscle mitochondria in *mdx* muscles, which is most susceptible to changes in DMD (Figure 3 and Figure 4). It is important to note that in this case we also revealed the normalization of the surface area of MAM-contacts between SR and interfibrillar mitochondria in the muscles of dystrophin-deficient animals (Figure 5 and Figure 6). An increase in the MAM-contact area in skeletal muscle is known to lead to a constant rise in mitochondrial Ca^2+^, which subsequently leads to increased ROS production and dysfunction of these organelles [24]. On the other hand, these structural changes in SR/mitochondria interactions may help maintain the activity of mitochondrial oxidative phosphorylation and the required level of ATP production. A decrease in MAM-contact formation apparently leads to a reduction in oxidative phosphorylation in organelles (Table 2) possibly due to complex changes in the calcium-dependent regulation of this process mediated by SR/mitochondria crosstalk and, at the same time, to a decrease in ROS production, which may have a therapeutic effect on the skeletal muscles of *mdx* mice. One could assume that the mitigation of oxidative stress in the skeletal muscles of *mdx* mice, as evidenced by a decrease in the level of MDA products (Figure 12C), may have an important effect on the intensity of degenerative processes. Indeed, antioxidants, particularly N-acetylcysteine (NAC), are known to reduce ROS concentrations in the muscles of *mdx* mice and improve their overall health [44]. However, the administration of NAC revealed side effects in in vivo experiments and was accompanied by a significant reduction in body weight gain and muscle weight of *mdx* mice [45]. It is important to note that we did not detect any effect of MTT on the body weight gain of *mdx* mice, which can be considered as an advantage of the approach. Also, it has recently been shown that the specific inhibition of mitochondrial VDAC channels by olesoxime suppresses the production of ROS by organelles, and this makes a significant contribution to alleviating the pathology of dystrophin-deficient skeletal muscles [17]. In addition, the mitochondria of *mdx*-MTT mice show a decrease in initial calcium overloading (Figure 12B), which may indicate that the excess of this ion is absorbed by additionally introduced mitochondria, preventing calcification of the muscle tissue of *mdx* mice (Figure 8B) and other dramatic effects of elevated Ca^2+^ concentrations. Importantly, this may be facilitated by an increase in the pool of subsarcolemmal mitochondria in the muscles of *mdx* + MTT mice (Figure 4C), whose defects contribute to dysregulation of Ca^2+^ homeostasis in the muscle fiber [46].

Currently the explanation of the mechanisms underlying the effects of MTT is quite speculative and even leading groups often do not set themselves such a task [6,10]. Our results suggest cautiously that MTT leads to the replenishment of functional mitochondrial proteins in the skeletal muscles of *mdx* mice, which generate less ROS and also normalizes interactions with mitochondria-related proteins and signaling molecules. It is also possible that exogenous mitochondria can act as scavengers that remove a dramatic excess of Ca^2+^ in dystrophin-deficient muscles. Moreover, we cannot exclude that the effect of MTT is due to external stimuli affecting the state of muscle tissue and mitochondria.

The *mdx* mice are characterized by the absence of embryonic lethality. This, among other things, may be due to the adaptation of the organism to the development of pathology in the form of a stable low level of the expression of genes of the mitochondrial signature, which allows for maintaining the minimum required level of metabolic activity and, at the same time, reducing the intensity of ROS production by dysfunctional mitochondria. In this case, a month-long MTT did not affect the expression of mitochondrial signature genes in the muscles of *mdx* mice. We did not note its effect on the reduced level of genes that control mitochondrial biogenesis, dynamics, and mitophagy (Figure 13). The mtDNA level reduced in muscles of *mdx* mice also did not change, despite encouraging electron microscopy data. Thus, MTT is not a trigger that affects the expression of nuclear genes that control mitochondrial networking.

Here we would like to note the limitations of this study. The major weakness of our study is the selection of control groups. In particular, it is necessary to study cross-injection effects (WT mitochondria to WT mice, *mdx* mitochondria to *mdx* mice, or *mdx* mitochondria to WT mice), since it can be assumed that mitochondria with a certain degree of dysfunction (like the mitochondria of *mdx* animals), acting as scavengers of negative factors (for example, excess calcium), may also have a positive effect. This will allow to evaluate the impact of unspecific effects of mitochondria injection on the phenotype.

Further studies are also needed to explore the following questions: (1) the concentration dependence of the introduction of mitochondria on the development of pathology; (2) the effects of the introduction of the various components (DNA, mitochondrial proteins and membranes) of mitochondria on the development of pathology; (3) testing the effects of mitochondria isolated from other tissues on the development of muscular dystrophy; (4) assessment of the therapeutic effect of MTT depending on the method of introducing mitochondria (intramuscular, intraperitoneal, into the bloodstream, etc.). It is also necessary to evaluate age-related effects (in most cases, earlier treatment is preferable, before the fourth week of development, demonstrating the onset of degenerative processes).

Both the duration of the positive effect and the overall long-term effects of MTT on the state of dystrophin-deficient skeletal muscles are currently unknown. It can be assumed that the effects of mitochondrial transfer are local since the expression of genes important for mitochondrial function has not been positively affected, and this may prohibit long-term DMD amelioration. A limitation may be a decrease in the intensity of the oxidative phosphorylation of mitochondria as a result of this procedure, which may reduce the pool of available ATP. Finally, it should be remembered that mitochondria are known to contain molecular motifs of damage-associated molecular patterns (DAMPs) that are capable of initiating a non-infectious inflammatory response [47], although in our case, the expression of genes associated with inflammation (*Tnf* and *Il6*) did not change in response to MTT (Figure 10).

The issue of the prospects of using MTT in the treatment of DMD in humans deserves special attention. It is known that humans show a more severe manifestation of DMD pathology compared to the rather mild phenotype of *mdx* mice, so the effectiveness of the approach needs to be carefully assessed, in particular, on more severe pathology models (like D2-*mdx* mice and others) and dystrophin-deficient biopsies. Another important issue is also the routes of administration of the organelles, as this may play an important role in the response to DAMPs. Furthermore, one of the central questions is the origin of donor mitochondria. Autografts (from one’s own non-pathological tissues), allografts, and xenografts can be considered as a source of mitochondria. The ethical and biological implications of each possible donor source should be considered.

## 5. Conclusions

The present work shows the effect of MTT on the development of muscle pathology in dystrophin-deficient *mdx* mice. The systemic intramuscular injections of allogeneic mitochondria lead to a decrease in the intensity of oxidative stress in the skeletal muscle mitochondria of *mdx* mice and a reduction in calcium overloading, which is also accompanied by the normalization of the organelle ultrastructure and SR/mitochondria contact interactions. This effect of MTT results in a reduction in degeneration/regeneration cycles, the level of tissue calcification in the skeletal muscles of *mdx* mice, the normalization of muscle fiber parameters, and the improved muscle strength and motor activity of the animals.

## Figures and Tables

**Table 1:** List of gene-specific primers for RT-PCR analysis.

Gene	Forward (5′→3′)	Reverse (5′→3′)
**Genes of inflammatory response**		
*Tnf* (Tumor necrosis factor-α)	CCACGTCGTAGCAAACCACC	ACAAGGTACAACCCATCGGC
*Il6* (interleukin-6)	GGGACTGATGCTGGTGACAA	TGCCATTGCACAACTCTTTTC
**Genes for mitochondrial dynamics**		
*Drp1* (Dynamin-related protein 1)	TTACAGCACACAGGAATTGT	TTGTCACGGGCAACCTTTTA
*Mfn2* (Mitofusin 2)	CACGCTGATGCAGACGGAGAA	ATCCCAGCGGTTGTTCAGG
**Gene for mitochondrial biogenesis**		
*Ppargc1a* (Peroxisome proliferator-activated receptor gamma coactivator 1-α)	CTGCCATTGTTAAGACCGAG	GTGTGAGGAGGGTCATCGTT
**Genes of mitophagy**		
*Pink1* (PTEN-induced kinase 1)	TTGCCCCACACCCTAACATC	GCAGGGTACAGGGGTAGTTCT
*Parkin*	AGCCAGAGGTCCAGCAGTTA	GAGGGTTGCTTGTTTGCAGG
**Genes used to estimate relative mtDNA levels**		
*Nd4* (NADH-ubiquinone oxidoreductase chain 4, mitochondrial)	ATTATTATTACCCGATGAGGGAACC	ATTAAGATGAGGGCAATTAGCAGT
*Gapdh* (Glyceraldehyde 3-phosphate dehydrogenase, nuclear)	GTGAGGGAGATGCYCAGTGT	CTGGCATTGCTCTCAATGAC
**Housekeeping gene**		
*Rplp2* (Ribosomal protein lateral stalk subunit P2)	CGGCTCAACAAGGTCATCAGTGA	AGCAGAAACAGCCACAGCCCCAC

**Table 2:** Parameters of respiration and oxidative phosphorylation of mouse skeletal muscle mitochondria.

Group	Respiration Rate, nmol O_2_/min per 1 mg of Protein				RCR
	State 2	State 3	State 4	State 3U_DNP_	
WT	19.7 ± 0.9	133.7 ± 2.8	32.3 ± 2.6	210.4 ± 8.3	4.2 ± 0.2
*mdx*	17.7 ± 0.8	121.6 ± 1.3 *	33.4 ± 1.8	178.6 ± 1.9 *	3.6 ± 0.1 *
*Mdx* + MTT	17.2 ± 0.9	105.2 ± 1.9 *#	31.4 ± 1.4	157.3 ± 1.8 *#	3.3 ± 0.1 *#

Mitochondria were energized by 2.5 mM glutamate and 2.5 mM malate. State 3 respiration was assessed by the addition of 200 μM ADP. The results are presented as means ± SEM (*n* = 8). * *p* < 0.05 versus control group (WT); # *p* < 0.05 versus *mdx* group.

## Data Availability

The data presented in this study are available upon request from the corresponding author.

## Supplementary Materials

The following supporting information can be downloaded at: https://www.mdpi.com/article/10.3390/biom14030316/s1, Figure S1: Typical curves of oxygen consumption by skeletal muscle mitochondria fueled by glutamate+malate in the absence (control) or presence of 10 μM cytochrome *c* (*cytC*); Figure S2: Representative histology images of gastrocnemius muscles (H&E staining) and the percentage of CNF in the gastrocnemius of experimental animals; Table S1: Parameters of respiration and oxidative phosphorylation of WT mice skeletal muscle mitochondria used for MTT.
