# Supplementary material for: Mitochondrial Transplantation Therapy Ameliorates Muscular Dystrophy in mdx Mouse Model
Source: Biomolecules. 2024 Mar 7;14(3):316. doi: 10.3390/biom14030316 (PMC10968431; doi:10.3390/biom14030316)
Supplement: Supplementary file 1 [file biomolecules-14-00316-s001.zip › biomolecules-2841336-supplementary.pdf]

biomolecules

MDPI

Article

Supplementary Material: Mitochondrial Transplantation Therapy Ameliorates Muscular Dystrophy in *mdx* Mouse Model

Mikhail V. Dubinin, Irina B. Mikheeva, Anastasia E. Stepanova, Anastasia D. Igoshkina, Alena A. Cherepanova, Alena A. Semenova, Vyacheslav A. Sharapov, Igor I. Kireev, Konstantin N. Belosludtsev

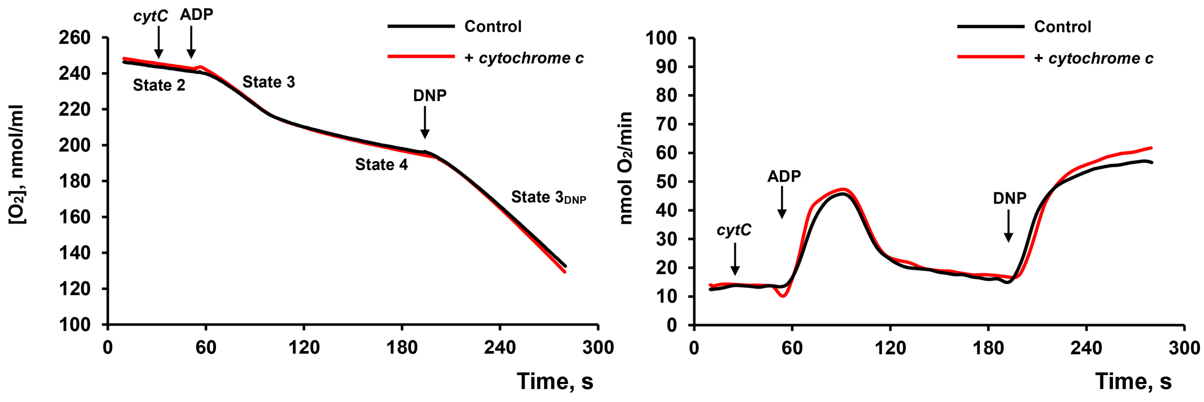

Fig. S1. Typical curves of oxygen consumption by skeletal muscle mitochondria fueled by glutamate+malate in the absence (control) or presence of 10 μM cytochrome *c* (*cytC*). Medium composition: 120 mM KCl, 5 mM NaH_2PO_4, 2.5 mM potassium malate, 2.5 mM potassium glutamate, and 10 mM HEPES-KOH (pH 7.4). Additions: 200 μM ADP, 50 μM DNP.

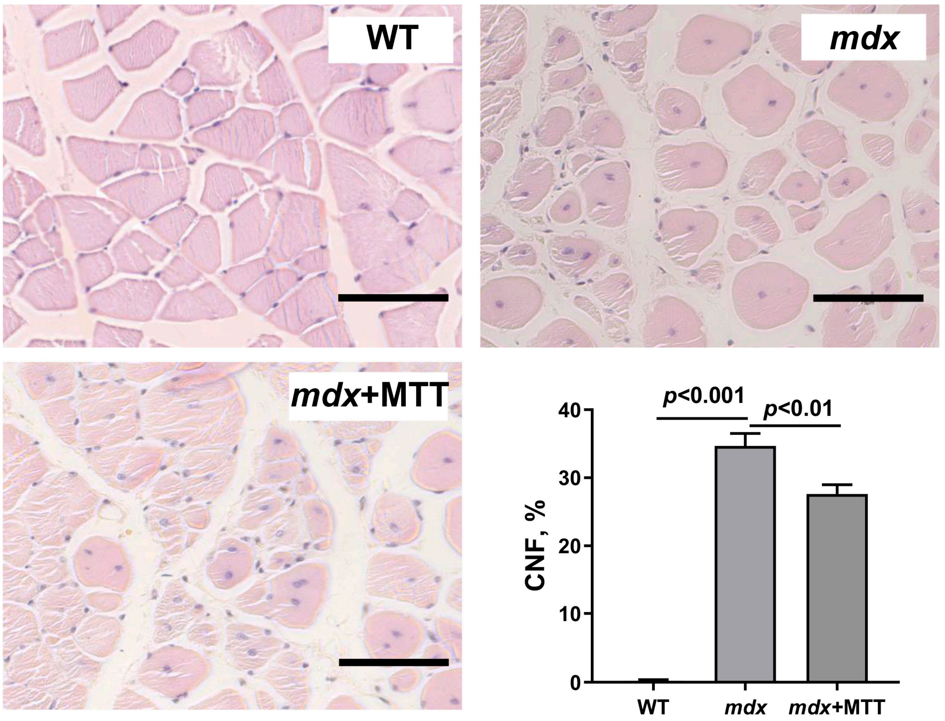

Fig. S2. Representative histology images of gastrocnemius muscles (H&E staining) and the percentage of CNF in the gastrocnemius of experimental animals. Scale bar is 75 μm. The data are presented as means ± SEM (*n*=4).

Table S1. Parameters of respiration and oxidative phosphorylation of WT mice skeletal muscle mitochondria used for MTT

**additions**	**V respiration, nmol O_2/min per 1 mg of protein**				**RCR**
	**State 2**	**State 3**	**State 4**	**State 3U$_{DNP}$**	
control	24.8±0.4	134.5±1.3	31.4±0.6	191.6±5.1	4.3±0.2
+ *cytC*	25.6±0.8	146.3±2.5*	33.3±1.3	211.4±4.3*	4.4±0.2

Mitochondria respiration was fueled by 2.5 mM glutamate and 2.5 mM malate. State 3 respiration was initiated by 200 μM ADP. The results are presented as means ± SEM (n = 20 (control) and n = 8 (*cytC*). * $p < 0.01$ versus control.
